# Selective sigma-2 ligands preferentially bind to pancreatic adenocarcinomas: applications in diagnostic imaging and therapy

**DOI:** 10.1186/1476-4598-6-48

**Published:** 2007-07-15

**Authors:** Hiroyuki Kashiwagi, Jonathan E McDunn, Peter O Simon, Peter S Goedegebuure, Jinbin Xu, Lynne Jones, Katherine Chang, Fabian Johnston, Kathryn Trinkaus, Richard S Hotchkiss, Robert H Mach, William G Hawkins

**Affiliations:** 1 Department of Surgery, Washington University School of Medicine, Saint Louis, USA; 2 Department of Anesthesiology, Washington University School of Medicine, Saint Louis, USA; 3 Alvin J. Siteman Cancer Center, Saint Louis, USA; 4 Department of Radiology, Washington University School of Medicine, Saint Louis, USA; 5 Division of Biostatistics, Washington University School of Medicine, Saint Louis, USA

## Abstract

**Background:**

Resistance to modern adjuvant treatment is in part due to the failure of programmed cell death. Therefore the molecules that execute the apoptotic program are potential targets for the development of anti-cancer therapeutics. The sigma-2 receptor has been found to be over-expressed in some types of malignant tumors, and, recently, small molecule ligands to the sigma-2 receptor were found to induce cancer cell apoptosis.

**Results:**

The sigma-2 receptor was expressed at high levels in both human and murine pancreas cancer cell lines, with minimal or limited expression in normal tissues, including: brain, kidney, liver, lung, pancreas and spleen. Micro-PET imaging was used to demonstrate that the sigma-2 receptor was preferentially expressed in tumor as opposed to normal tissues in pancreas tumor allograft-bearing mice. Two structurally distinct sigma-2 receptor ligands, SV119 and WC26, were found to induce apoptosis to mice and human pancreatic cancer cells *in vitro *and *in vivo*. Sigma-2 receptor ligands induced apoptosis in a dose dependent fashion in all pancreatic cell lines tested. At the highest dose tested (10 μM), all sigma-2 receptor ligands induced 10–20% apoptosis in all pancreatic cancer cell lines tested (p < 0.05). In pancreas tumor allograft-bearing mice, a single bolus dose of WC26 caused approximately 50% apoptosis in the tumor compared to no appreciable apoptosis in tumor-bearing, vehicle-injected control animals (p < 0.0001). WC26 significantly slowed tumor growth after a 5 day treatment compared to vehicle-injected control animals (p < 0.0001) and blood chemistry panels suggested that there is minimal peripheral toxicity.

**Conclusion:**

We demonstrate a novel therapeutic strategy that induces a significant increase in pancreas cancer cell death. This strategy highlights a new potential target for the treatment of pancreas cancer, which has little in the way of effective treatments.

## Background

Pancreas cancer is the fourth most common cause of cancer-related mortality in the United States[1]. There has been little progress in the management of this disease and the annual mortality rate remains nearly identical to the annual incidence rate. There were an estimated 37,000 deaths attributable to this disease in the United States in 2006[1]. The five-year survival for patients diagnosed with pancreas cancer is only 4%. Poor outcome is the result of difficulty in achieving early diagnosis and failure of modern treatments including surgery, radiation and chemotherapy. Normally, chemotherapy and radiation therapy for pancreatic cancer is palliative. For localized disease surgery remains the single most effective therapy, however even with complete surgical resection, the prognosis remains dismal and the majority of patients recur with systemic metastases. Therefore there is a critical need for an effective systemic therapy utilizing a new strategy.

Programmed cell death or apoptosis is an essential process in cell homeostasis and eukaryotic development[2,3]. Recent studies have shown that failure of apoptosis is both central to the evolution of cancer and related to resistance toward modern adjuvant treatment such as chemo- or radiation therapies [4-6]. Therefore, there is considerable interest in targeting the molecular pathways of apoptosis as a component of cancer therapy.

We and others have recently reported that intracellular delivery of pro-apoptotic peptides can induce apoptosis in cancer cells *in vitro *and in animal models of adenocarcinomas *in vivo *[7-9]. For example, we have studied TAT-Bim a dual domain molecule comprised of an internalization motif (the polybasic region of HIV-1 TAT) and a biological effector domain (the BH3 domain of Bim) which leads to apoptosis. To demonstrate anti-tumor effectiveness *in vivo*, the peptides were injected intra-tumorally due to the non-specific cell permeation capacity of the TAT peptide[10]. Direct intra-tumoral injection of the TAT-Bim compound effectively reduced tumor size and prolonged survival in animal models of pancreatic cancer[9]. In order to translate these promising anti-cancer approaches into clinically viable therapies, cancer cell-specific targeting is required. The targeting strategy or domain can then be combined with biological effector domain to achieve cancer cell-specific death with preservation of normal tissues.

Several strategies have been developed to target delivery of therapy to tumors. (1) Physical targeting with either directed radiation[11] or intratumoral injection[9]. (2) Receptor-directed targeting with antibodies (for example, Herceptin[12], rituximab[13]) or ligands (for example, TRAIL[14]). (3) Prodrugs that are activated by enzymes active in the local milieu (for example, MMP-9[15]). (4) Differences in metabolic needs (for example, Gleevec[16,17]). Each of these approaches has inherent limitations, for example physical targeting has no ability to treat distant metastases and the antibody- or ligand-based targeting approaches are restricted to portions of the tumor that are accessible to macromolecules.

In this study we have begun to explore whether small molecules that selectively accumulate in cancer cells may provide yet another approach to targeted therapeutic delivery. A number of small molecules have been used in whole-body imaging studies and preferentially accumulate in tumors. One such class of small molecules is the family of sigma-2 receptor ligands.

The sigma-2 receptor has been characterized as a molecule that is highly expressed in rapidly proliferating cells and down-regulated when cells become quiescent[18]. Sigma-2 receptor expression has been reported in various human cancer cells such as breast[19], brain[20], bladder[21], colon and melanoma[22]; however, no studies have been reported on sigma-2 receptor expression in pancreas adenocarcinoma. Sigma-2 receptor-specific ligands have been found to induce apoptosis [19-22], raising the possibility that these ligands can act as sensitizers to standard chemotherapies.

Here we extend the body of evidence that the sigma-2 receptors are generic cancer markers by demonstrating that the sigma-2 receptor are over-expressed in pancreatic cancer cell lines *in vitro *and in Panc-02 tumor allografts established in C57Bl/6 mice *in vivo*. We further demonstrate that sigma-2 receptor ligands induce apoptosis in pancreatic cancer cells via a caspase-3 dependent pathway. And finally we show that systemic treatment with sigma 2 receptor-specific ligands slows the growth of established tumors in allograft murine models of pancreatic cancer without disrupting organ function.

## Results

### Sigma-2 receptor expression *in vitro *and *in vivo*

Murine pancreatic tumor cells (Panc-02) and human pancreas cancer cell lines (AsPC-1, CFPAC-1, Panc-1) bound high levels of the sigma-2 receptor ligands *in vitro *(Figure 2A and 2B). Sigma-2 receptor ligands had minimal binding to single cell suspensions prepared from most normal murine tissues including kidney, liver, lung, and brain. Cells from spleen and pancreas exhibited higher binding capacity for sigma-2 receptor ligands at the highest dose tested (100 nM) and the spleen appeared to contain two populations of cells with regard to sigma-2 receptor ligand binding; however, even compared to the high KO5-138-binding population of splenocytes, approximately 10-fold more KO5-138 bound per cell to the syngeneic Panc-02 tumors grown in vivo (Figure 2B). These findings translated into the ability to image the tumor in vivo using micro-PET (Figure 2C).

**Figure 1:**
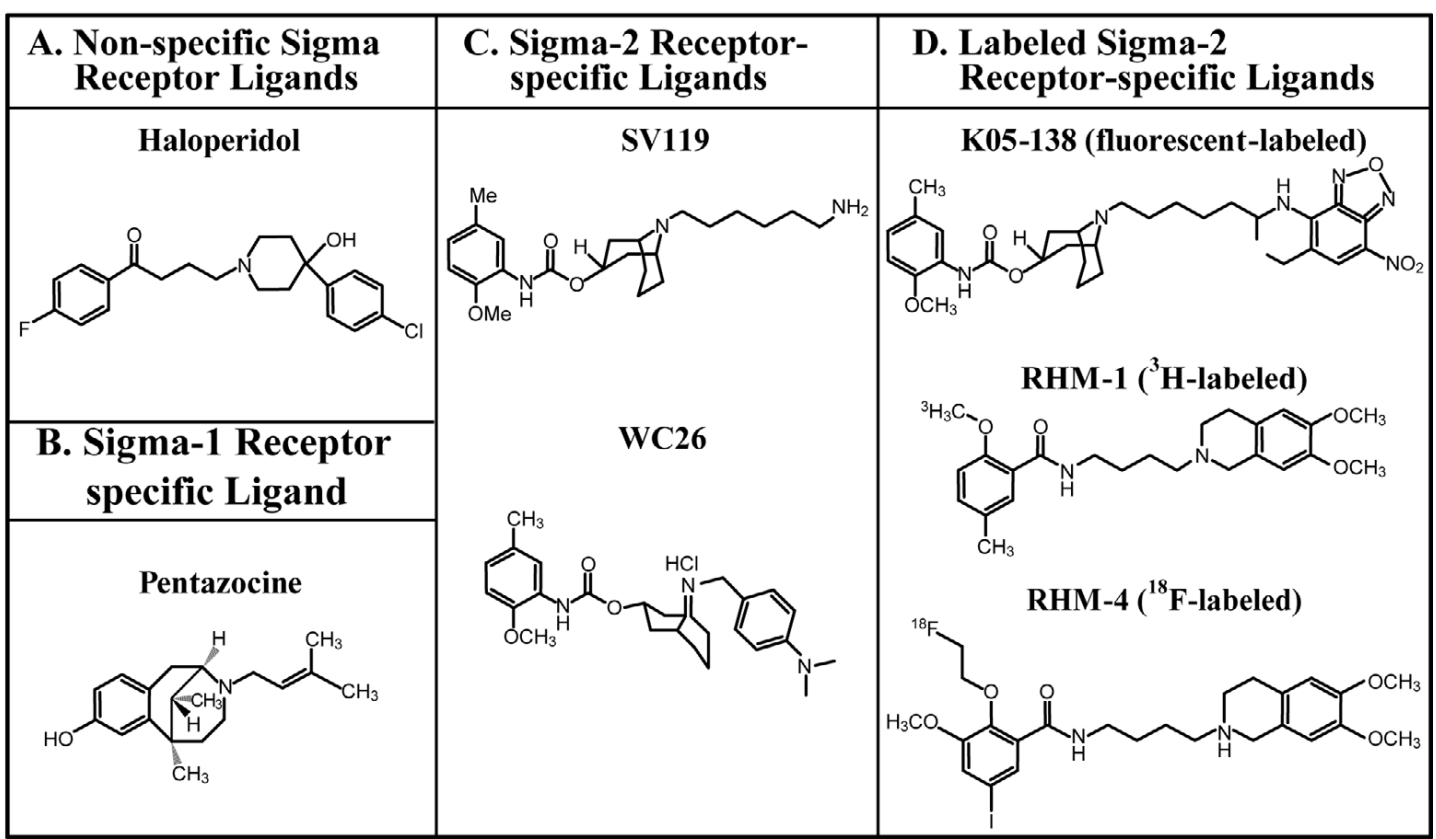
**Structure of sigma ligands utilized in this paper**. Panel A: Haloperidol is a non-specific sigma-1 and sigma-2 receptor ligand. Panel B: Pentazocine is a sigma-1 specific ligand. Panel C: Sigma-2 receptor-specific ligands SV119 and WC26. Panel D: Chemically-labeled sigma-2 receptor-specific ligands K05-138 (fluorescein-labeled), RHM-1 (^3^H-labeled), and RHM-4 (^18^F-labeled).

**Figure 2:**
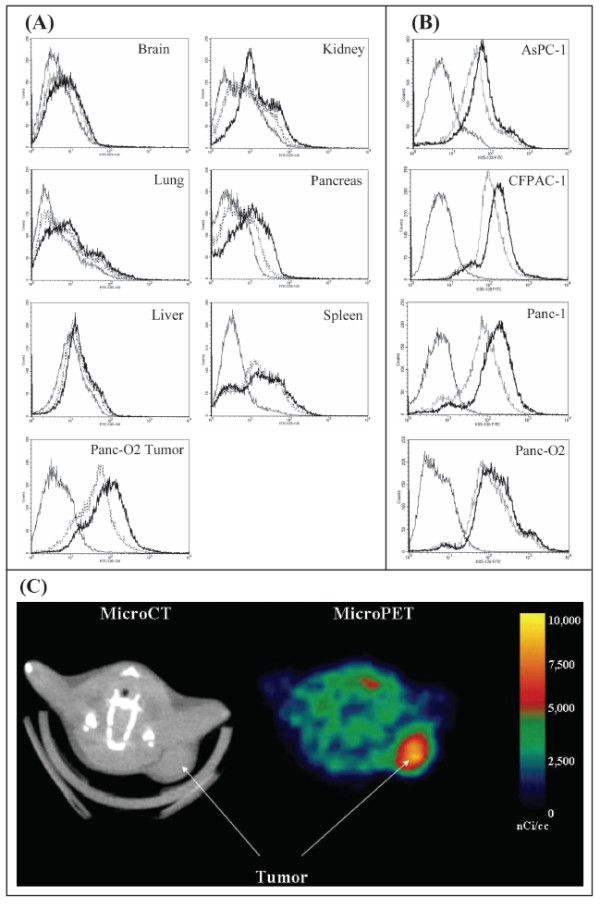
**Sigma-2 ligands preferentially bind to cancer as opposed to normal tissues**. **(A): **Single cell suspensions were prepared from liver, lung, pancreas, brain, kidney, spleen and pancreatic tumors (panc-02 bearing mice). Single cell suspensions were incubated for 1 hour with KO5-138 (a fluorescent labeled sigma-2 receptor ligand, at 50 nM (thin doted line) or 100 nM (thick solid line) of ligand, or left unstained (thin solid line). FACS histograms demonstrate 100 fold more florescence in tumors compared to normal tissues. **(B)**: Human pancreatic cancer lines (CFPAC-1, Panc-1, AsPC-1) demonstrate similar high degree of sigma-2 ligand binding to when compared to our murine model (Panc-02). Fluorescein signal peaks were shown in unstained control (thin solid line), 50 nM of ligand (dotted line), or 100 nM of ligand (thick solid line). The experiment was repeated twice with identical results. **(C)**: Representative Micro PET/CT images of pancreas adenocarcinoma (Panc-02) bearing C57BL/6 mice administered RHM-4 (^18^F-labled sigma-2 ligand). The tumor indicated by arrows was approximately 1 cm^3^. The additional hot spot represents metastatic tumor in a regional lymph node.

### Binding assays

**Figure 3:**
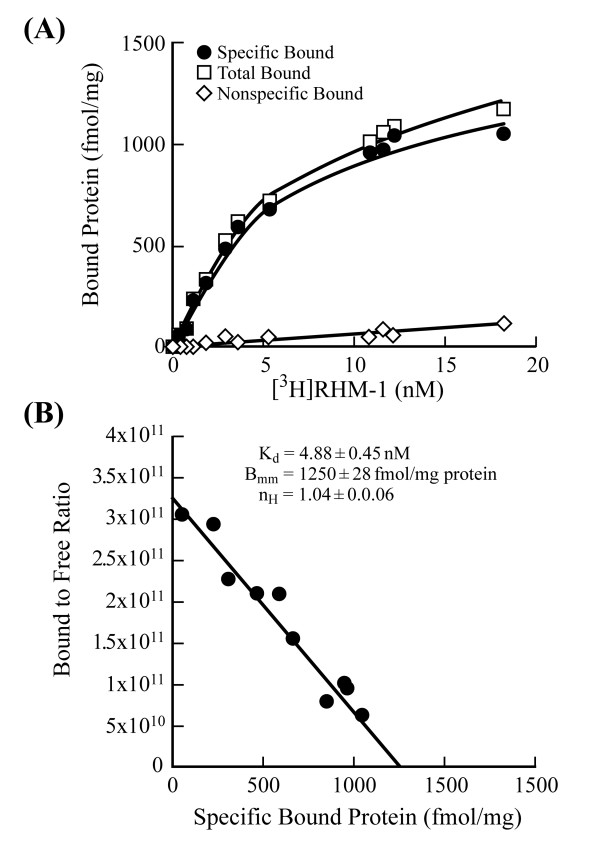
**Scatchard analysis of [^3^H]RHM-1 binding to the sigma-2 receptors in membrane homogenates from Panc-02 tumor allografts. **(A): Representative saturation binding experiments which show the total bound, non-specific bound and specific bound. (B): Representative Scatchard plots which were used to determine *K*_d _and *B*_max _values.

Direct saturation binding studies were carried out using [^3^H]RHM-1 with membrane homogenates of PancO2 tumor allografts. Based on Scatchard analysis, the *K*_d _and *B*_max _values of the receptor-radioligand binding of [^3^H]RHM-1 were 4.88 nM and 1250 fmol/mg protein (Figure 3). The mean of *n*_H _values was close to unity, suggesting that the receptor binding of [^3^H]RHM-1 displayed one-site and non-cooperative binding characteristics.

### Sigma-2 receptor ligand induces caspase-3/7 dependent cancer cell apoptosis

**Figure 4:**
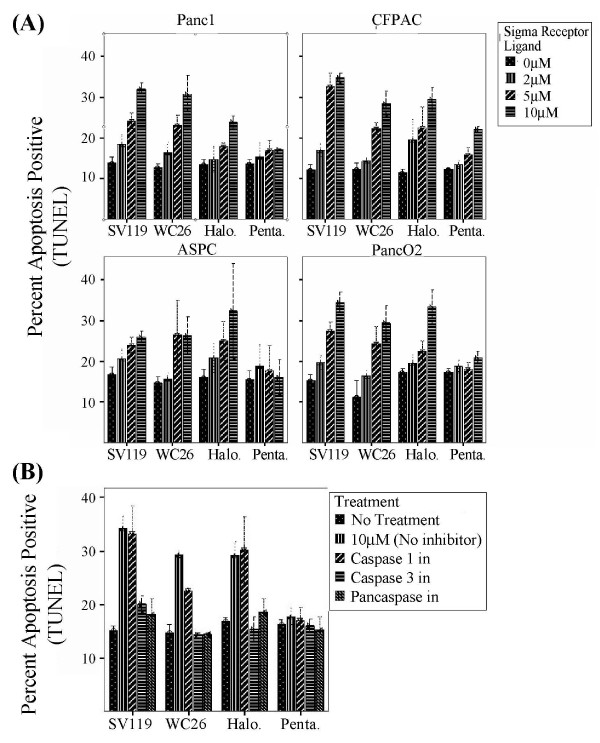
**Sigma-2 receptor ligands induce apoptosis of pancreatic cancer cells via a caspase 3 dependent pathway. (A): **Treatment with WC26, SV119 and haloperidol (Halo.) caused dose-dependent apoptosis in all pancreatic cancer cell lines tested. At the highest dose tested, all sigma-2 receptor ligands resulted in significant apoptosis (25–35%, p < 0.05). **(B): **Sigma-2 related apoptosis was prevented by pan-caspase inhibitor (Z-VAD-fmk) and caspase-3/7 inhibitor (DEVD-CHO), while the caspase-1 inhibitor (YVAD-CHO) had no effect (Panc-02 cells).

Treatment with sigma-2 specific ligands (WC26 and SV119) and the sigma-1/sigma-2 promiscuous ligand haloperidol caused dose-dependent apoptosis in all pancreas cancer cell lines tested. All cell lines tested had a background rate of 10–15% apoptosis over the incubation period. At the highest dose tested (10 μM), all sigma-2 receptor ligands induced an additional 10–20% of the cells to undergo apoptosis (Figure 4A). The sigma-1 receptor-specific ligand pentazocine had minimal toxicity to all cell lines tested with the exception of CFPAC-1 which exhibited an 8–10% increase in apoptosis at the highest dose tested. The pan-caspase inhibitor (Z-VAD-fmk) and caspase-3/7 inhibitor (DEVD-CHO) prevented sigma-2 receptor ligand-induced apoptosis in panc-02 cells, while the caspase-1 inhibitor (YVAD-CHO) had no effect (Figure 4B). Similar results were seen with human pancreatic cancer cell lines (AsPC-1, CFPAC-1, Panc-1, data not shown).

### *In vivo *treatment with a sigma-2 receptor ligand causes tumor apoptosis with minimal effects on normal tissues

**Figure 5:**
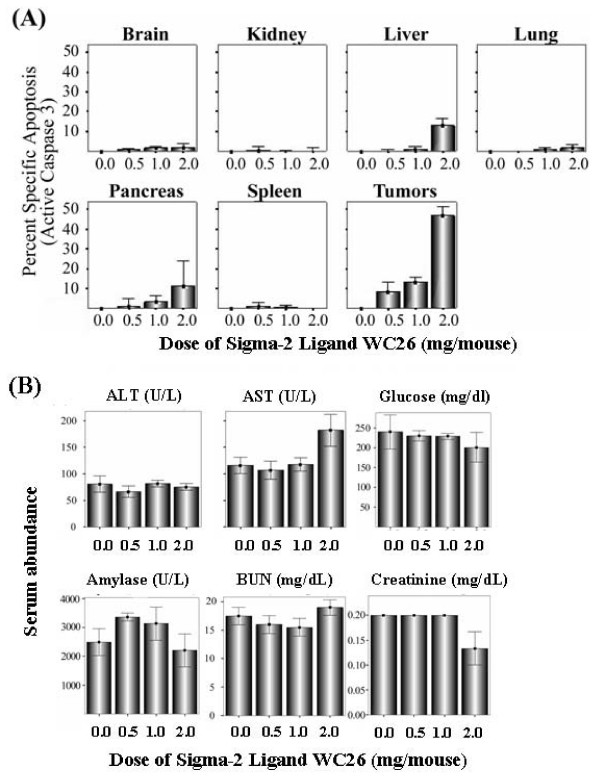
**Systemic administration of Sigma-2 receptor ligand induces substantial tumor apoptosis without major toxicity. (A): **Systemic administration of the sigma-2 receptor ligand WC26 induces considerable apoptosis in Panc-02 tumor allografts (up to 50% of tumor cells were active caspase-3 positive following a single 2 mg dose of WC26, p < 0.0001). Data reported as percent specific apoptosis (percent specific apoptosis = observed apoptosis – tissue specific background apoptosis). **(B): **The mice appeared normal and no apparent toxicity was noted in serum biochemical analysis or by immunohistochemistry (data not shown). Reference ranges for the blood chemistry panel in C57Bl/6 mice: ALT: 18–184 U/L, AST: 55–251 U/L, glucose: 174–335 mg/dL, amylase: 2595 +/- 212 U/L, BUN: 34–58 mg/dL, creatinine < 1.1 mg/dL.

Systemic administration of the sigma-2 receptor ligand WC26 did not induce apoptosis in brain, lung, kidney or spleen at any of the concentrations tested as measured by FACS for active caspase-3 (Figure 5A). There was a small amount of apoptosis (<10%) in the pancreas and livers of these animals while their tumors had dose-dependent increases in apoptosis (up to 50% of tumor cells were active caspase-3 positive following a single 2 mg dose of WC26, p < 0.0001). The mice appeared normal and no apparent toxicity was noted in serum biochemistry data (Figure 5B). There was a modest effect at the highest dose of WC26 tested with elevated AST and depressed creatinine and glucose levels, though these values are still within their reference ranges [23].

### Sigma-2 receptor ligand therapy slows tumor growth and confers a survival advantage *in vivo*

**Figure 6:**
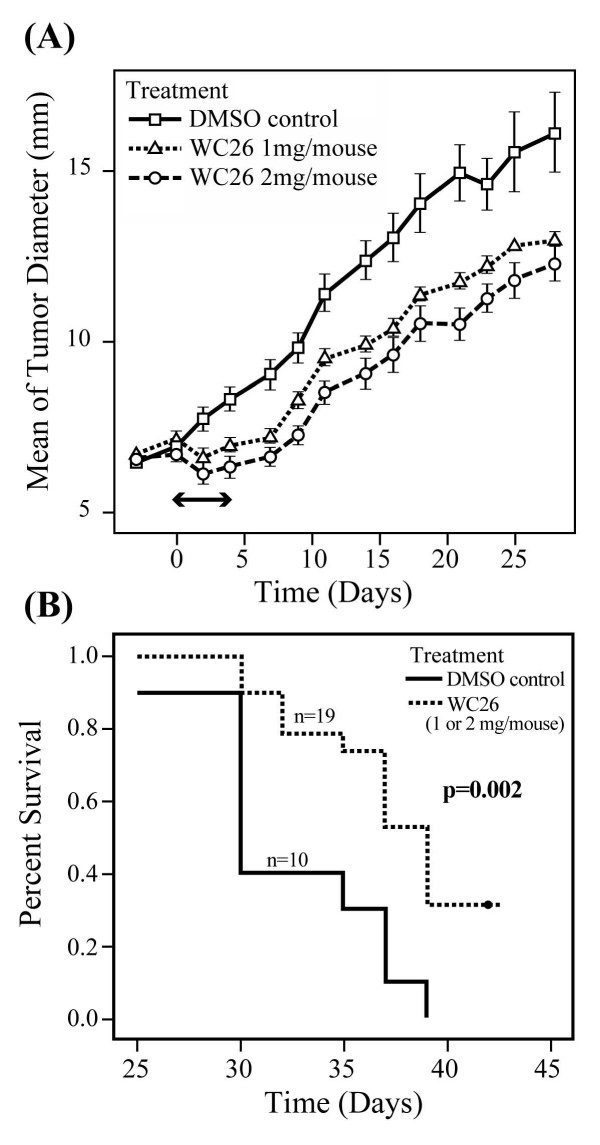
**Systemic administration of sigma-2 receptor ligand slows tumor growth and improves survival**. Mice with established pancreas tumors were treated with 5 days of WC26 in one of two dose ranges (2 mg/day, n = 9; Dash line or 1 mg/day n = 10; dashed line) or vehicle control (20% DMSO n = 10; solid line). **(A): **Mice treated with WC26 had smaller tumors compared to animals vehicle control (p < 0.0001). The two treatment groups were not statistically different. The double-headed arrow denotes the treatment period (5 days). **(B): **Survival of mice treated with WC26 compared favorably to the mice treated vehicle control (p= 0.002). Survival endpoints were defined as tumor diameter >15 mm or tumor ulceration.

The anti-tumor properties of sigma-2 receptor ligand (WC26) monotherapy were evaluated *in vivo *in an established syngeneic tumor model using Panc-02 cells in C57BL/6 mice. Once tumors were established (5–8 mm diameter), mice were randomized into one of three treatment groups: daily intraperitoneal injection of 50 or 100 mg/kg of WC26 or vehicle control (20% DMSO, 200 μl) once a day for five days. In both WC26 treatment groups, tumors shrank during and immediately following treatment while tumors in the vehicle-treated animals continued to grow (Figure 6A). Although regrowth occurred in the Sigma-2 ligand treatment groups after treatment was stopped, there was a significant delay compared to control animals. Using tumor burden of 15 mm or ulceration as a surrogate endpoint for survival, we found a significant survival benefit in the Sigma-2 receptor ligand treatment group (Figure 6B; p = 0.002). Based on blood chemistry parameters, there was no acute, systemic toxicity observed in any of the treated animals. In addition, no acute toxicity was observed on immunohistochemical evaluation of normal tissues after WC26 treatment (data not shown).

## Discussion

We and others have shown that sigma 2 receptor-specific ligands such as WC26 have both diagnostic and therapeutic potential. Although the sigma 2 receptor has not been identified, some characterization has occurred. It is an approximately 20 kDa protein exclusively found in lipid rafts throughout membrane encapsulated cellular compartments, yet it has resisted sequencing efforts. Pharmacologic studies suggest that the sigma 2 ligand binding site is well conserved between rodents and primates, and we have demonstrated that sigma 2 receptor-specific ligands induce apoptosis in both mouse and human pancreas adenocarcinoma cell lines equally.

Apoptosis is a crucial mechanism for cellular homeostasis and failure of this mechanism is central to the development of cancer and associated with resistance toward adjuvant treatment such as chemo- or radiation therapies[5,24]. Overexpression of anti-apoptotic proteins including Bcl-2 family members and AKT/PKB pathway agonists have been found in a variety of human cancers including pancreas[25], breast[26], prostate [27] and lung[28]. As a result, there is considerable interest in targeting the intracellular apoptotic apparatus for cancer therapy.

Sigma-2 receptor ligands have recently been reported to induce apoptosis in several tumor cells *in vitro *[19-22]. Here we reported that sigma-2 receptor is highly expressed in human and mouse pancreatic cancer cells, and sigma-2 receptor-selective ligands induce tumor apoptosis in a caspase-3 dependent manner. Mouse and human pancreatic cancer cell lines as well as established Panc-02 tumor allografts established in C57Bl/6 mice expressed the sigma-2 receptor at significantly higher levels than normal mouse tissues, and the selective accumulation of the sigma-2 receptor ligand in the tumor could be used to image the tumor *in vivo*. These findings suggest that sigma-2 receptor ligands have potential utility for diagnostic imaging of pancreatic cancer extending the spectrum of their utility beyond what has previously been reported for breast cancer and supporting the hypothesis that the sigma-2 receptor is a generic marker for cellular proliferation [18,29-31].

Targeting the sigma-2 receptor may also have utility in cancer treatment. Ligands to the sigma-2 receptor (WC26, SV119, and haloperidol) caused dose-dependent apoptosis in all tumor cell lines *in vitro*, while the sigma-1 receptor-selective ligand pentazocine did not cause cell death in three of four pancreatic cancer cell lines. Interestingly the CFPAC cell line underwent dose dependent cell death in the pentazocine treatment group. Sigma-1 antagonists have been shown to induce apoptosis[32], suggesting that CFPAC cells may have a different defect in their apoptotic program.

The mechanism of sigma-2 receptor ligand-induced cell death has not been established. Previous studies have reported that sigma 2 receptor-specific ligands induce apoptosis in a p53- and caspase-independent manner by causing oxidative stress and increasing the concentration of intracellular ceramide [33,34]. Other groups have shown that the caspase-dependent mitochondrial pathway is activated in response to treatment with sigma 2 receptor-specific ligands [35]. Our results show that inhibition of the executioner caspases abrogates WC26-induced apoptosis in pancreas adenocarcinoma cell lines, suggesting that sigma 2 receptor-specific ligand-induced apoptosis may be cell type and/or ligand specific. Pancreas cancer cells incubated with sigma-2 receptor ligands were found to have active caspase-3 within 4–6 hours and DNA fragmentation within 20 hours (data not shown). Furthermore, pan-caspase and caspase-3/7 specific inhibition rescued sigma-2 receptor ligand-treated cells from death. Our data do not clarify whether sigma-2 receptor-specific ligand-induced cell death occurs predominantly by the mitochondrial (intrinsic) or receptor-mediated pathway (extrinsic).

Our results are consistent with previous reports describing cancer cell-selectivity of sigma-2 receptor ligands as well as the ability of these molecules to cause caspase-dependent cell death (apoptosis) both *in vitro *and *in vivo*. By establishing that pancreas cancer cell lines and Panc-02 tumor allografts established in C57Bl/6 mice respond similarly to sigma-2 receptor ligands, we have extended the utility of these molecules and are demonstrating the generality of targeting the sigma-2 receptor for both diagnostic and therapeutic applications. Importantly, we showed that 24 hours after a single dose of WC26 there was significant tumor apoptosis *in vivo *with minimal apoptosis in normal tissues. More apoptosis was observed in Panc-02 cells grown *in vivo *than in the same cell line grown *in vitro*. This result is likely due to the up to 20-fold difference in concentrations of the sigma 2 receptor-specific ligand between these experiments, but could also be due to differential sensitivity of the Panc-02 cells when grown in 2-dimensional culture compared to the 3-dimensional architecture adopted *in vivo*. Although a small amount of caspase-3 activity was seen in normal liver and pancreas in dissociated tissues, this finding was not recapitulated in an immunohistochemical study of normal tissues after WC26 treatment (data not shown). Furthermore, there were no apparent, systemic side effects in the WC26 treated animals (Figure 5B).

In addition to causing tumor cell apoptosis *in vivo *with minimal peripheral toxicity, sigma-2 receptor ligand treatment of tumor-bearing mice led to tumor stability and regression in some animals. Although all tumors regrew shortly after treatment was stopped, WC26-treated tumors remained smaller and fewer ulcerated than tumors in vehicle-injected control animals. Systemic therapy with WC26 was successful in reducing tumor mass and/or altering the course of tumor growth after therapy. If patients with locally advanced pancreatic cancer respond similarly to treatment with sigma-2 receptor ligands they might become candidates for pancreatic cancer treatment.

## Conclusion

Using small molecule probes we have demonstrated that the sigma-2 receptor is selectively over-expressed on pancreas cancer cells both *in vitro *and *in vivo*. We have also shown proof of principle that this finding can be exploited for both diagnostic imaging and therapeutic use. We have identified the sigma-2 receptor as a potential target for the treatment of pancreas cancer; selectively triggering or sensitizing tumor cells toward apoptosis.

## Materials and methods

### Sigma receptor compounds and chemicals

Sigma-1 and sigma-2 receptor ligands (Figure 1) were synthesized as previously described [36]. Caspase inhibitors (ZVAD-FMK, YVAD-CHO, and DEVD-CHO) were obtained from Calbiochem (San Diego, CA). Unless otherwise specified, all other materials were obtained from Sigma-Aldrich (Saint Louis, Missouri, USA).

### Cell lines

Mouse pancreatic cancer (Panc-02) and several human pancreatic cell lines (CFPAC-1, AsPC-1, and Panc-1) were maintained in supplemented RPMI containing glutamine (2 mmol/L), pyruvate (1 mmol/L), penicillin and streptomycin (100 IU/mL), and 10% FBS. All cell culture processes were carried out in a humidified atmosphere of 5% CO2 at 37°C.

### Sigma-2 expression study *in vitro*

Lung, liver, spleen, kidney, pancreas, brain and established tumor from C57BL/6 mice were minced to 1-mm in size and digested in a RPMI buffer containing 1 mg/ml collagenase (Sigma-Aldrich, St. Louis, MO) and 0.1 mg/ml DNase (Sigma-Aldrich, St. Louis, MO) for 45 min to obtain a single-cell suspensions. After filtering, contaminating erythrocytes were lysed in Ammonium Chloride (ACK) buffer, pelleted and resuspended in PBS (pH 7.4). Single cell suspensions were incubated for 1 hour with KO5-138 (a fluorescent labeled sigma-2 receptor ligand, Figure 1) at 50 nM or 100 nM of ligand, left unstained. All lines were then washed 3 times with PBS, and evaluated by Fluorescence Activated Cell Sorting (FACS).

### Binding assays (Scatchard analysis)

The tritiated compound [^3^H]RHM-1 was synthesized by American Radiolabeled Chemicals, Inc. (St. Louis, MO) via O-alkylation of the corresponding phenol precursor as previously described[37]; chemical purity was greater than 99% and the specific activity of the radioligand was 80 Ci/mmol. Membrane homogenates were prepared from ~400 mg Panc-02 mouse tumor allografts, which were removed from tumor bearing mice and frozen on dry ice immediately and stored at -80°C until used. Before homogenization, the tumor allografts were allowed to thaw slowly on ice. Tissue homogenization was carried out at 4°C using a Potter-Elvehjem tissue grinder at a concentration of 1 g of tissue/ml of 50 mM Tris-HCl at pH 8.0. The crude membrane homogenate was then transferred to a 50 ml centrifuge tube and resuspended to a concentration of 200 mg of tissue/ml of 50 mM Tris-HCl. Additional homogenization was accomplished using an Ultra-Turrax T8 polython homogenizer (IKA Works, Inc, Wilmington, NC). The final homogenate was then centrifuged for 10 min at 1000 g, the pellet discarded and the supernatant mixed by vortexing and stored at -80°C until used. The protein concentration of the suspension was determined (DC protein assay, Bio-Rad, Hercules, CA) and averaged ~10 mg of protein/ml of stock solution. Approximately 200 μg membrane homogenates were diluted with 50 mM Tris-HCl buffer, pH 8.0 and incubated with [^3^H]RHM-1 in a total volume of 150 μl at 25°C in 96 well polypropylene plates (Fisher Scientific, Pittsburgh, PA). The concentrations of the radioligand ranged from 0.1–18 nM. After incubation of 60 min, the reactions were terminated by the addition of 150 μl of cold wash buffer (10 mM Tris-HCl, 150 mM NaCl, pH 7.4, at 4°C) using a 96 channel transfer pipette (Fisher Scientific, Pittsburgh, PA), and the samples harvested and filtered rapidly to 96 well fiber glass filter plate (Millipore, Billerica, MA) that had been presoaked with 100 μl of 50 mM Tris-HCl buffer, pH 8.0 for 1 hour. Each filter was washed with 200 μl of ice-cold wash buffer for a total of three washes. A Wallac 1450 MicroBeta liquid scintillation counter (Perkin Elmer, Boston, MA) was used to quantitate the bound radioactivity[30], non-specific binding was determined from samples which contained 10 μM RHM-1. The equilibrium dissociation constant (*K*_d_) and maximum number of binding sites (*B*_max_) were determined by a linear regression analysis of the transformed data using the method of Scatchard[38].

Data from saturation radioligand binding studies was transformed to determine the Hill coefficient, *n*_H_, defined as:

log⁡BsBmax⁡−Bs=log⁡Kd+nHlog⁡L
 MathType@MTEF@5@5@+=feaafiart1ev1aaatCvAUfKttLearuWrP9MDH5MBPbIqV92AaeXatLxBI9gBaebbnrfifHhDYfgasaacH8akY=wiFfYdH8Gipec8Eeeu0xXdbba9frFj0=OqFfea0dXdd9vqai=hGuQ8kuc9pgc9s8qqaq=dirpe0xb9q8qiLsFr0=vr0=vr0dc8meaabaqaciaacaGaaeqabaqabeGadaaakeaacyGGSbaBcqGGVbWBcqGGNbWzdaWcaaqaaiabdkeacnaaBaaaleaacqWGZbWCaeqaaaGcbaGaemOqai0aaSbaaSqaaiGbc2gaTjabcggaHjabcIha4bqabaGccqGHsislcqWGcbGqdaWgaaWcbaGaem4CamhabeaaaaGccqGH9aqpcyGGSbaBcqGGVbWBcqGGNbWzcqWGlbWsdaWgaaWcbaGaemizaqgabeaakiabgUcaRiabd6gaUnaaBaaaleaacqWGibasaeqaaOGagiiBaWMaei4Ba8Maei4zaCMaemitaWeaaa@4D33@

*B*_s _is the amount of the radioligand bound specifically; *L *is the concentration of radioligand. *n*_H_, Hill slope, was determined from Hill plot of log⁡BsBmax⁡−Bs
 MathType@MTEF@5@5@+=feaafiart1ev1aaatCvAUfKttLearuWrP9MDH5MBPbIqV92AaeXatLxBI9gBaebbnrfifHhDYfgasaacH8akY=wiFfYdH8Gipec8Eeeu0xXdbba9frFj0=OqFfea0dXdd9vqai=hGuQ8kuc9pgc9s8qqaq=dirpe0xb9q8qiLsFr0=vr0=vr0dc8meaabaqaciaacaGaaeqabaqabeGadaaakeaacyGGSbaBcqGGVbWBcqGGNbWzdaWcaaqaaiabdkeacnaaBaaaleaacqWGZbWCaeqaaaGcbaGaemOqai0aaSbaaSqaaiGbc2gaTjabcggaHjabcIha4bqabaGccqGHsislcqWGcbGqdaWgaaWcbaGaem4Camhabeaaaaaaaa@3C8A@ versus log*L*.

### Sigma-2 expression study *in vivo*

MicroPET (positron emission tomography)/CT Imaging was performed to confirm the uptake of the sigma-2 receptor ligand after injection of [18F]_4 _labeled Sigma-2 ligand; RHM-4 in tumor bearing mice. Briefly, female C57Bl/6 mice were implanted subcutaneously in the nape of the neck with Panc-02 mouse pancreatic adenocarcinoma cells (1.0 × 10^6 ^cells in 200 μl RPMI) 7–10 days before the study date. Average tumor burden on the day of imaging was ~1.0 cm^3^. The animals were injected with of [18F]_4 _labeled Sigma-2 ligand via tail vein and imaged at 2 hours after injection.

### Evaluation of cytotoxicity of sigma-2 ligands *in vitro*

Tumor cells were seeded at a density of approximately 0.2 × 10^6 ^cells per well in 12-well plates in 1.0 ml culture medium. Cells were split and pre-incubated at 37°C in humidified 5% CO_2 _for more than 24 hours (Panc-02) and 48 hours (CFPAC-1, AsPC-1, Panc-1) to insure uniform growth conditions. Compounds were dissolved in DMSO and added to the culture medium at the concentrations indicated. The final concentration of DMSO in the cell culture medium was less than 1%. The cells were then incubated for 24 hours at 37°C in humidified 5% CO_2_. The extent of apoptosis was subsequently measured as previously reported[9]. Briefly, staining was performed on trypsin-EDTA treated cultures that had been fixed with 1% paraformaldehyde and 90% methanol. Cell pellets were resuspended in TUNEL reagent (APO-BRDU kit, San Diego, CA) or cleaved caspase-3 antibody (Cell Signaling Technology, Inc. Boston, MA) and incubated overnight at room temperature (TUNEL) or 4°C (cleaved caspase-3). After washing, cells were resuspended in fluorescein antibody or 7-AAD buffer and incubated for 1 hour at room temperature. Cell-associated fluorescence was determined using a flow cytometry (FACScan, BD Biosciences) and analyzed with CellQuest software (BD Biosciences).

### Antitumor effect of sigma-2 receptor ligand *in vivo*

All studies were performed in accordance with an animal protocol approved by the Washington University Institutional Animal Care Facility. Female C57BL/6 mice (8–12 weeks old) were purchased from the NCI and acclimated for at least 1 week before tumor implantation. All mice were injected in the right flank with 200 μl of a single cell suspension containing 1.0 × 10^6 ^Panc-02 cells. Treatment of the tumors started 2 weeks after tumor implantation when their size reached a mean diameter of 5–8 mm. To evaluate the effect of sigma-2 receptor ligands both systemically and on tumor *in vivo*, several mice were sacrificed after a single treatment. Necropsy was performed and single cell suspensions were prepared from retrieved organs. The extent of apoptosis in these cells was measured by FACS (described above). For the survival study, mice (N = 10 per group) were treated with sigma-2 receptor ligand at the stated concentration or vehicle control once a day for 5 days. Mean tumor diameter was measured three times each week. All mice were euthanized when the tumors reached a mean diameter of 15 mm or when the tumors ulcerated [39].

### Statistical analysis

For *in vivo *experiments, Kaplan-Meier survival curves were plotted and differences were compared using a log-rank test. Tumor sizes and FACS results were analyzed using linear mixed repeated measures models. Hypothesis tests were corrected for multiple testing using a Hochberg step-up procedure. A *p*-value of less than 0.05 was considered significant for all analyses.

## Competing interests

The author(s) declare that they have no competing interests.

## Authors' contributions

HK: Performed experiments, interpreted results, drafted manuscript

JEM: Drafted manuscript, critical revision to manuscript, designed experiments, interpreted results

POS: Performed experiments, drafted manuscript, critical revision to manuscript

PSG: Performed survival studies, critical revision to manuscript

JX: Performed binding studies

LJ: Performed imaging studies

KC: Designed and conducted experiments

FJ: Performed experiments

KT: Statistical review

RSH: Critical revision to manuscript, designed experiments, interpreted results.

RHM: Synthesis of sigma-2 ligands, imaging studies

WGH: Designed experiments, interpreted results, final draft of manuscript

All authors have read and approved the final manuscript.
